# Noninvasive Ventilation Strategies in Neonates

**DOI:** 10.1007/s13312-025-00077-7

**Published:** 2025-04-29

**Authors:** Jogender Kumar, Praveen Kumar, Vineet Bhandari

**Affiliations:** 1 Neonatal Unit, Department of Pediatrics, Post Graduate Institute of Medical Education and Research, Chandigarh, 160012 India; 2 Division of Neonatology, Department of Pediatrics, Cooper Medical School of Rowan University, The Children’s Regional Hospital at Cooper, One Cooper Plaza, Camden, NJ 08103 USA

**Keywords:** Preterm, RDS, NCPAP, NIPPV, HHHFNC, NIV-NAVA, NHFV, NIV

## Abstract

We provide recommendations on neonatal noninvasive ventilation (NIV) strategies used in the delivery room (DR) and neonatal intensive care unit (NICU). A systematic search was performed in the PubMed, Embase, and CENTRAL databases to identify relevant literature from the past 5 years. A critical review of the available literature was conducted to provide context-specific recommendations. In the DR, we recommend using nasal continuous positive airway pressure (NCPAP) or nasal intermittent positive pressure ventilation (NIPPV) with a T-piece resuscitator (TPR). Surfactant replacement therapy should be administered early (< 2 h of life) in infants requiring NCPAP of 6–7 cm H_2_O and FiO_2_ > 0.3, using less invasive surfactant administration techniques. Infants should be transported to the NICU on positive pressure support using NCPAP or TPR. In extremely preterm infants with severe respiratory distress requiring intubation in the DR, surfactant should be considered during the intubation. If equipment and expertise are available in the NICU, NIPPV is the preferred mode of NIV. Nasal masks or short binasal prongs are the preferred nasal interfaces. A heated, humidified, high flow nasal cannula is not recommended as the primary mode of NIV. Additional clinical trials are needed for nasal high frequency ventilation and noninvasive ventilation neurally adjusted ventilatory assist modes of NIV. Guidelines for the recommended initial and maximal settings for primary, post-extubation, and weaning off NIV in neonates are provided in this article. NIPPV and NCPAP are the preferred modes of NIV in neonates with respiratory distress.

## Introduction

Overall, 1–1.5% of all neonates develop respiratory distress syndrome (RDS), requiring respiratory support [1]. Respiratory distress is the most common indication for neonatal intensive care unit (NICU) admission, accounting for up to 80% of neonatal admissions [1], with RDS as a leading cause of mortality and morbidity. Recently, noninvasive ventilation (NIV) has become the first-line approach in managing neonatal RDS, with invasive ventilation becoming a reserve or backup mode among those who fail NIV or where it is unlikely to be effective or preferably avoided. Prematurity is the leading cause of respiratory distress, and with the improved survival of preterm infants, thorough, up-to-date knowledge of neonatal ventilation strategies is of utmost importance. In this article, we review the current evidence on neonatal NIV approaches from the delivery room (DR) until successful weaning from NIV.

## Evidence Acquisition

We searched PubMed, Embase, and CENTRAL for relevant original articles, systematic reviews, and evidence-based guidelines on various aspects of neonatal NIV. This included keywords related to the population (neonate, infant, preterm), intervention (continuous positive airway pressure or CPAP, nasal intermittent positive pressure ventilation or NIPPV, surfactant, oxygen), and outcomes (extubation, ventilation days, bronchopulmonary dysplasia, or BPD). The search was limited to the last 5 years (2019–2024) unless there was no update in the past five years. We also searched the guidelines issued by various societies [American Academy of Pediatrics (USA) or AAP, National Institute for Health and Care Excellence (UK) or NICE, National Neonatology Forum (India) or NNF, European Standards of Care for Newborn Health or ESCNH, etc.] on relevant topics. All relevant retrieved articles were collated, critically analyzed, appraised, and summarized according to the objective of the review. We first evaluated existing evidence-based most recent guidelines from prominent societies and most recent systematic reviews and meta-analyses of randomized clinical trials (RCTs) to provide the most up-to-date evidence. We searched for evidence pertaining to low- and middle-income countries (LMICs), such as India, to make it context-specific. We provide a summary of the most recent evidence in Table 1. In situations in which evidence did not exist, the authors provided expert opinions.

**Table 1:** Evidence summary for noninvasive ventilatory support in neonates

Authors/Comparison	Number of participants (Studies)	Outcomes	Effect estimate RR (95% CI)	Certainty of evidence	Remarks
*Respiratory support in the delivery room*					
Tribolet et al. (2023) T-piece Resuscitator versus Self-Inflating Bag for Neonatal Resuscitation [2]	1268 (5 RCTs)	BPD	0.68 (0.48–0.96)		Most data compare a T-piece with a self-inflating bag
	1266 (4 RCTs)	Need for Intubation	0.72 (0.58–0.88)		
	1197 (3 RCTs)	Mechanical ventilation	0.8 (0.67–0.96)		
*Comparison of primary noninvasive respiratory support modalities in preterm infants*					
Lemyre et al. (2023) NIPPV *vs*. NCPAP as primary respiratory support for preterm infants [25]	1958 (17 RCTs)	Respiratory failure	0.65 (0.54–0.78)	Moderate	No difference in mortality, IVH, and air leaks A considerable proportion of infants were born at > 30 weeks
	1848 (16 RCTs)	Need for intubation	0.67 (0.56–0.81)	Moderate	
	1284 (12 RCTs)	BPD	0.70 (0.52–0.92)	Low	
Hodgson et al. (2023) Nasal high flow (HHHFNC) *vs* nasal CPAP in preterm infants [29]	2042 (9 RCTs)	Treatment failure within 72 h	1.70 (1.41–2.06)	Moderate	Significantly less nasal trauma (RR 049, 0.36 to 0.68)
	1917 (8 RCTs)	BPD	1.14 (0.74–1.76)	Low	
	2009 (9 RCTs)	Mortality	0.78 (0.44–1.39)	Low	
Kuitunen et al. (2024) NIV-NAVA versus NCPAP [27]	183 (3 RCTs)	Intubation	0.91 (0.56–1.48)	Low	No difference in BPD and other outcomes
	183 (3 RCTs)	Surfactant	0.85 (0.56–1.29)	Low	
Abdel-Latif et al. (2024) Nasal HFV vs NCPAP [31]	531 (4 RCTs)	Mortality	1.00 (0.41–2.41)	Very low	No difference in BPD and IVH
	571 (5 RCTs)	Endotracheal intubation	0.52 (0.33–0.82)	Low	
Hodgson et al. (2023) Nasal high flow (HHHFNC) *vs* NIPPV [29]	343 (4 RCTs)	Treatment failure within 72 h	1.27 (0.90–1.79)	Moderate	Significantly less nasal trauma (RR 0.21, 0.09 to 0.47)
	271 (3 RCTs)	BPD	1.19 (0.66–2.12)	Low	
	254 (3 RCTs)	Mortality	0.78 (0.36–1.69)	Low	
Abdel-Latif et al. (2024) Nasal HFV vs NIPPV [31]	307 (5 RCTs)	BPD	0.63 (0.42–0.95)	Low	No difference in mortality, MV, IVH
Abdel-Latif et al. (2024) Nasal HFV vs Invasive ventilation [31]	80 (1 RCT)	Mortality	0.67 (0.20–2.18)	Very low	No difference in mortality or other outcomes
Silveira et al. (2023) LISA vs INSURE for surfactant administration [11]	1599 (14 RCTs)	Need for Invasive Ventilation	0.60 (0.47–0.76)		There is slight heterogeneity in the method of LISA
	1758 (13 RCTs)	BPD	0.65 (0.51–0.82)		
	1093 (9 RCTs)	Air leaks	0.60 (0.38–0.96)		
	1558 (13 RCTs)	Mortality	0.76 (0.58–1.00)		
	170 (1 RCT)	Any grade IVH	1.77 (1.06–2.96)	Very low	
*Comparison of Secondary (Post-extubation) respiratory support modalities in preterm infants*					
Ho JJ et al. (2024) NCPAP vs No CPAP as post-extubation support in preterm infants [20]	726 (9 RCTs)	Extubation failure	0.62 (0.51–0.76)	Low	No difference in mortality, ROP, and severe IVH
	726 (9 RCTs)	Reintubation	0.79 (0.64–0.98)	Very low	
	92 (1 RCT)	BPD	0.89 (0.47–1.68)	Very low	
Lemyre et al. (2023) NIPPV vs NCPAP [35]	2738 (19 RCTs)	Extubation Failure	0.75 (0.67–0.84)	Moderate	No difference in mortality and BPD
	2404 (13 RCTs)	Air leak	0.57 (0.37–0.87)	Low	
Martins et al. (2022) HHHFNC versus NCPAP in preterm infants [30]	751 (5 RCTs)	Nasal Trauma	OR 0.21 (0.08–0.52)		No difference in death, BPD, and other outcomes
	448 (4 RCTs)	Extubation failure	OR 1.33 (0.67–2.63)		
Abdel-Latif et al. (2024) Nasal HFV vs NCPAP [31]	1897 (11 RCTs)	Extubation Failure	0.42 (0.35–0.51)	Low	No difference in death or composite outcome
	1829 (10 RCTs)	BPD	0.78 (0.67–0.91)	Low	
Kuitunen et al. (2024) NIV-NAVA versus NCPAP/NIPPV [27]	96 (2 RCTs)	Extubation Failure	0.29 (0.10–0.81)	Very low	One study was on CPAP and another used NIPPV in the control arm
Abdel-Latif et al. (2024) Nasal HFV vs NIPPV [31]	1364 (6 RCTs)	Extubation Failure	0.69 (0.54–0.89)	Moderate	No difference in mortality, BPD, and IVH

*BPD* bronchopulmonary dysplasia, *CI* confidence interval, *CPAP* continuous positive airway pressure, *HFV* high frequency ventilation, *HHHFNC* heated humidified high flow nasal cannula, *INSURE* intubation, surfactant administration and extubation, *IVH* intraventricular hemorrhage, *LISA* less invasive surfactant administration, *MV* mechanical ventilation, *NCPAP* nasal continuous positive airway pressure, *NIPPV* nasal intermittent positive pressure ventilation, *NIV-NAVA* noninvasive ventilation neurally adjusted ventilatory assist, *OR* odds ratio, *RCT* randomized controlled trial, *PS* pressure support, *RDS* respiratory distress syndrome, *ROP* retinopathy of prematurity, *RR* risk ratio.

## Results

### Noninvasive Ventilation in the Delivery Room

Effective DR respiratory support is critical for neonates born preterm with respiratory distress, to ensure adequate oxygenation and to prevent brain and lung injuries. The cornerstone of neonatal resuscitation is the timely initiation of effective positive pressure ventilation (PPV), which is recommended for newborns who are not breathing or have inadequate respiratory effort at birth. In most LMICs, a self-inflating bag (SIB) is used to provide PPV at birth [2]. However, it subjects a premature fragile lung to uncontrolled barotrauma and volutrauma and lacks positive end-expiratory pressure (PEEP), subjecting them to additional atelectotrauma. If the infant does not breathe spontaneously, a T-piece resuscitator (TPR), which is a simple yet effective “gentle lung ventilation” device that provides controlled peak inspiratory pressure (PIP) and PEEP, is recommended. It requires minimal training and has significant potential for use in LMICs, where DR ventilators are rarely available. A meta-analysis of RCTs comparing TPR with SIB showed that TPR reduced the need for intubation in DR, duration of PPV, need for mechanical ventilation (MV), surfactant use, and risk of BPD [2]. The initial recommended pressure for T-piece resuscitation is a PEEP of 5–8 cm H_2_O and a PIP of 20–25 cm H_2_O [3]. An oxygen blender is recommended in the DR to titrate the fraction of inspired oxygen (FiO_2_) during resuscitation and thereafter. The International Liaison Committee on Resuscitation (ILCOR) recommends an initial FiO_2_ of 0.21 to 0.30 for preterm infant resuscitation with subsequent titration as per minute-wise oxygen saturation by pulse oximetry (SpO_2_) targets [4]. Recently, an updated individual participant data meta-analysis reported lower mortality in those who received high initial FiO_2_ (≥ 0.90) for resuscitation in preterm infants born at < 32 weeks of gestation [5]. This observation requires confirmation in large prospective trials.

In preterm infants with respiratory distress, maintenance of PEEP reduces alveolar collapse and helps establish and maintain functional residual capacity (FRC), which subsequently reduces the work of breathing. Early initiation of nasal CPAP (NCPAP) in the DR itself compared to intubation and MV has been shown to be associated with a significant reduction in the composite outcome of death and/or BPD [6]. The ILCOR and European consensus guidelines recommend that spontaneously breathing preterm infants with respiratory distress in DR should be stabilized and managed with NCPAP rather than intubation [4, 7]. Routine intubation of extremely preterm infants in the DR for PPV has fallen out of favor. Intubation and MV should be reserved for infants who do not respond to noninvasive positive pressure support.

For infants who require endotracheal intubation and MV soon after birth, early surfactant therapy (< 2 h of life) should be considered [7]. The NNF guidelines strongly suggest considering surfactant for neonates < 28 weeks gestation if they are intubated in the DR for severe respiratory distress [8]. If the infant is spontaneously breathing on NCPAP but has worsening respiratory distress, then it is recommended to administer the surfactant once FiO_2_ > 0.3 and NCPAP pressure reaches ≥ 6 cm H_2_O to maintain SpO_2_ [7, 9]. However, others have suggested surfactant administration only if the FiO_2_ is ≥ 0.4 [10], especially beyond 2 h of life [9]. Recently, bedside lung ultrasound (LUS) has gained popularity for determining surfactant administration, and future trials might suggest an individualized approach based on the LUS score [7]. In non-intubated, spontaneously breathing infants, less invasive surfactant administration (LISA) has become a favored method for surfactant administration in developed countries [11]. Because of its potential to reduce the need for MV (along with BPD and air leaks) [11] and potentially cost-effective option in resource-limited settings, it has also gained popularity in LMICs [12]. According to a survey in 2022 from India, nearly 70% of surveyed units use LISA as one of the methods for surfactant administration [12], of which nearly one-third use it as a preferred method.

During the entire period, close monitoring of the use of humidified gases and blended oxygen is recommended. FiO_2_ should be titrated to ensure that SpO_2_ is within the target range [7]. Preterm infants (< 32 weeks gestational age or GA) requiring positive pressure support should be started on caffeine therapy, preferably within a few hours to the first 48 h after birth [7, 9, 13]. Early caffeine administration is associated with a reduction in BPD in preterm infants [13].

### Ventilation During Transport

Infants who spontaneously breathe with minimal respiratory distress can be transported using a low flow nasal cannula (e.g., using nasal prongs). However, those requiring NCPAP in DR should be transported to the NICU on positive pressure support through NCPAP or TPR.

### Noninvasive Ventilation in the NICU

#### Nasal Continuous Positive Airway Pressure

NCPAP refers to the application of continuous distending pressure (CDP) to the lungs through nasal passages. NCPAP helps maintain FRC and prevents atelectasis. Bench studies have shown that NCPAP decreases airway resistance, improves pulmonary compliance, increases endogenous surfactant production, improves gas exchange, stabilizes the airway, and decreases the work of breathing [14]. Based on the method of CPAP generation (constant/variable), various devices (bubble CPAP, infant flow driver, flow-driven conventional ventilators, high frequency devices, etc.) have been used to generate PEEP. Multiple RCTs have compared the efficacy and safety of different NCPAP delivery systems but have failed to provide conclusive evidence [6, 14]. A recent meta-analysis suggested that bubble CPAP may lead to a lower incidence of CPAP failure, compared with other CPAP modes, without affecting BPD and mortality [15]. Considering its ease of use, cost-effectiveness, and slightly better efficacy, bubble CPAP may be the preferred device for NCPAP [7, 14, 15].

Regarding nasal interfaces*,* nasopharyngeal prongs and nasal endotracheal tubes have gone out of favor owing to their disadvantages. Currently, the most common nasal interfaces for CPAP delivery in LMICs are short binasal prongs (SBP), nasal masks, and the RAM^®^ cannula. Nasal injury is seen in 30–50% of neonates receiving NCPAP and is commonly affected by the nasal interface, humidification, and the CPAP device, along with expertise in nursing care [16]. A recent Cochrane review comparing nasal masks with SBP suggested that nasal mask use may reduce NCPAP failure (risk ratio (RR) 0.72, 95% confidence interval (CI) 0.58 to 0.90) and moderate to severe nasal injury (RR 0.55, 95% CI 0.44 to 0.71) [17]. However, continuous use of nasal masks requires dedicated nursing care with a good nurse-to-patient ratio, which is challenging in most settings in LMICs, such as India. Therefore, multiple studies have compared various strategies to reduce nasal trauma, including rotation of nasal masks with SBP [16]. A recent meta-analysis suggested that periodic rotation of nasal masks and SBP might be superior to using SBP alone and might be used in settings where continuous use of nasal masks remains challenging owing to limited nursing resources [16].

Recently, the RAM^®^ cannula has gained popularity in India. The RAM^®^ cannula (Neotech, Valencia, CA, USA) is a class-1 medical device for delivering oxygen. However, because of its softness, ease of use, comfort, cost-effectiveness, and relatively low nasal injury rates, it is frequently used to deliver NCPAP and NIPPV [18]. Research indicates that the actual pressure delivered through the RAM cannula is often lower than the set pressure, especially when compared to SBP [19]. However, a recent meta-analysis comparing RAM^®^ cannula with SBP observed a significant reduction in moderate to severe nasal injury without any significant effect on CPAP/NIPPV failure [18], which makes it a potential alternative to SBP.

In conclusion, evidence suggests that nasal masks are the most effective and safest nasal interfaces for delivering NIV. However, in our experience, thorough knowledge and experience of the given interface in the unit, along with diligent nursing care, is crucial to reducing nasal injury, irrespective of the interface used (Box 1). Table 1 provides a summary of the interventions.

NCPAP can be used as a primary and post-extubation support in spontaneously breathing preterm infants. As stated earlier, evidence suggests that starting early CPAP therapy prevents respiratory failure in preterm infants [6]. Therefore, NCPAP should be started early in infants with respiratory distress. The guidelines for the initial NCPAP settings and their titrations are shown in Table 2.

**Table 2:** Suggested typical settings for primary and post-extubation modes of noninvasive ventilation in preterm infants*

Respiratory support	Initial settings	Titration	Suggested maximum settings
*Primary respiratory support*			
Nasal CPAP	PEEP: 5 cm of H_2_O Flow: 5 L/min	Adjust as per chest inflation on radiograph or degree of intercostal retractions	PEEP: 8 cm H_2_O (Some people use up to 10 cm H_2_O with intensive monitoring, though evidence is lacking supporting this approach)
NIPPV	PIP: 15–16 cm H_2_O PEEP: 5–6 cm H_2_O Ti: 0.5 s Rate: 40/min Target MAP: 8–10 cm of H_2_O	PEEP: as per chest inflation on radiograph or degree of intercostal retractions PIP: As per chest rise and auscultation	PIP: 22–25 cm H_2_O PEEP: 8 cm H_2_O MAP: 14–16 cm H_2_O FiO_2_: 0.6
HHHFNC	Flow: Preterm 2–3 L/min Term: 3–5 L/min	Titrate the flow as per distress. Do not increase flow beyond 8 L/min	Flow: Preterm: 5–6 L/min Term: 8 L/min
Nasal HFV	MAP: 8–10 cm H_2_O Amplitude: 20 to 25 cm H_2_O Frequency: 6 to 8 Hz Ti: 50%	Adjust MAP as per chest inflation on a radiograph Amplitude as per pCO_2_ levels	Max: 10–12 cm H_2_O
*Post-extubation support*			
Nasal CPAP	PEEP: 7–8 cm H_2_O Flow: 5 L/min	Adjust as per chest inflation on radiograph or intercostal retraction	8–10 cm H_2_O (Higher PEEP is useful in established BPD) FiO_2_: 0.6
NIPPV	PIP: 16–18 cm H_2_O PEEP: 6–7 cm H_2_O Ti: 0.5 s Rate: 40/min Target MAP: 10–12 cm of H_2_O	PEEP: as per chest inflation on radiograph or degree of intercostal retractions PIP: As per chest rise and auscultation	PIP: 25 cm H_2_O PEEP: 8 cm H_2_O MAP: 13–14 cm H_2_O FiO_2_: 0.6
Nasal HFV	MAP: 9–10 cm H_2_O Amplitude: 20 to 25 cm H_2_O Frequency: 8 to 9 Hz Ti: 50%	Adjust MAP as per chest inflation on a radiograph Amplitude as per pCO_2_ levels	

^*^These are generic settings as a clinical guide; they should be individualized based on the clinical condition and postmenstrual age

*BPD* bronchopulmonary dysplasia, *CPAP* continuous positive airway pressure, *FiO*_*2*_ fraction of inspired oxygen, *HFV* high frequency ventilation, *HHHFNC* heated humidified high flow nasal cannula, *MAP* mean airway pressure, *NIPPV* nasal intermittent positive pressure ventilation, *pCO*_*2*_ partial pressure of carbon dioxide, *PEEP* positive end-expiratory pressure, *PIP* peak inspiratory pressure, *Ti* inspiratory time

NCPAP is a popular post-extubation support method for preterm infants. It is better to extubate preterm infants (particularly < 32 weeks) to NCPAP rather than room air alone, as the former is known to reduce the risk of extubation failure and reintubation [19]. Recent NNF India clinical practice guidelines and European guidelines recommend NCPAP administration as the primary respiratory support for all preterm neonates with RDS [7, 20]. The level of post-extubation support depends on the lung condition. There is some evidence that extubation to higher NCPAP (9–11 cm H_2_O) compared to standard pressures (6–8 cm H_2_O) might reduce extubation failure in extremely premature infants [21].

#### NCPAP for Transient Tachypnea of Newborn (TTN)

CPAP helps in TTN by facilitating the clearance of lung fluid and preventing alveolar collapse. There is very limited data on NCPAP use in TTN. NCPAP has been shown to decrease the duration of tachypnea [mean difference (95% CI): − 21.10 h (− 22.9 to − 19.3); 1 RCT, 64 neonates] [22]. No significant differences were observed in the need for MV or mortality. Although useful in premature infants, term and near-term neonates with TTN often do not tolerate NCPAP. Therefore, the use of NCAP should be limited to those with moderate to severe respiratory distress.

### NCPAP for Meconium Aspiration Syndrome (MAS)

Meconium aspiration can lead to partial or complete airway obstruction, atelectasis, surfactant inactivation, and persistent pulmonary hypertension. Early NCPAP might help reduce atelectasis, preserve surfactant, and maintain FRC. Two RCTs compared NCPAP with oxygen alone [25, 26]. A trial enrolling neonates with moderate to severe respiratory distress reported that CPAP resulted in a decreased risk of invasive MV (IMV) [Odds ratio (95% CI): 0.09 (0.02–0.43)] [24]. However, another recent trial enrolling neonates with mild to moderate disease did not find any significant difference in MV or other outcomes [23]. These findings suggest that infants with mild disease can be managed using oxygen alone, whereas those with moderate to severe distress should preferentially be managed with NCPAP.

### Nasal Intermittent Positive Pressure Ventilation

NIPPV is a mode of NIV in which a set number of intermittent breaths with PIP are delivered over and above the PEEP. It might be delivered in synchronization with neonates’ breathing efforts (termed synchronized NIPPV, hereafter referred to as SNIPPV) or independent of respiratory efforts (non-synchronized NIPPV). Compared to NCPAP, mandatory intermittent PIP helps to increase tidal and minute volumes and hence improves gas exchange. A trial of NIPPV can also be useful for neonates with recurrent apnea. A recent meta-analysis of 17 RCTs suggested that NIPPV may be superior in reducing the need for intubation and possibly reducing the incidence of BPD compared to NCPAP as a primary respiratory support in preterm infants with respiratory distress [25]. Similarly, as post-extubation support, NIPPV has been shown to be superior to NCPAP in reducing extubation failure and the need for reintubation in preterm infants; however, there was no difference in BPD or mortality [25]. Although evidence suggests that NIPPV might be better than NCPAP as primary as well as post-extubation support, its universal availability remains a challenge in LMICs like India. Therefore, NNF guidelines suggest that preterm very low birth weight neonates with respiratory distress should be initiated on either NCAP or NIPPV. NIPPV may preferably be used in preterm neonates with very low birth weight, where equipment and expertise are available (conditional recommendation) [8].

Observational studies suggest that owing to asynchrony between neonatal respiratory efforts and ventilator-delivered breaths, the pressure delivered to the alveolar level in non-synchronized NIPPV is much lower than the set pressure [26]. Thus, if inflation is synchronized with the infant’s inspiratory efforts, the pressure delivered might be higher. Pneumatic capsules (Graseby capsules) and neurally adjusted ventilatory assists (NAVA) are commonly used for synchronization. Evidence suggests that ventilator-generated SNIPPV might be better than non-synchronized NIPPV, particularly as post-extubation support [26, 27], albeit limited by the small sample size and significant heterogeneity among studies. Furthermore, a recent meta-analysis comparing NIV-NAVA with NCPAP as the primary respiratory support did not find any significant differences in BPD, respiratory failure, or mortality [27]. Most of the included studies were conducted in high-income countries, limiting their generalizability. The availability of the device, cost, and expertise remains a major concern in LMICs such as India. Therefore, with current evidence, SNIPPV may not be used beyond clinical trials [8]. A recent safety study of a novel bubble NIPPV device showed promise as a potential low-cost option [28]; however, RCTs for clinical efficacy are still needed for definitive recommendations.

### Heated Humidified High Flow Nasal Cannula (HHHFNC)

HHHFNC provides heated and humidified air at 2–8 L/min via the nostrils through a specialized NC. Recently, HHHFNC has gained popularity among healthcare providers as an alternative to NCPAP. It is postulated to work by various mechanisms, including washout of the nasopharyngeal dead space, providing variable distending pressure, and optimal gas conditioning [13, 29]. A major reason for its increased acceptance in neonatal units is its ease of use, reduced risk of nasal trauma, better tolerance in infants (particularly above 1.5 kg), ease of performing Kangaroo Mother Care, and improved mother-infant bonding [29]. Multiple trials have compared HHHFNC with NCPAP as primary [29] and post-extubation support [30]. These trials consistently showed that HHHFNC use was associated with significantly less nasal trauma; however, there was a trend toward increased respiratory failure. Since its non-inferiority compared with NCPAP has not been established, current evidence does not support the routine use of HHHFNC as primary or post-extubation support in preterm infants (particularly at < 28 weeks) [13, 30].

### Nasal High Frequency Ventilation

Nasal HFV (NHFV) is a relatively new mode of NIV. This mode is rarely used in LMICs and there is limited evidence from high-income settings. In this mode, extremely small tidal volumes at a high frequency superimposed on continuous gas flow are delivered through nasal prongs/masks. It is postulated to improve alveolar ventilation and active carbon dioxide (CO_2_) expiration. A recent meta-analysis comparing NHFV with NCPAP as primary respiratory support suggested that NHFV may reduce the need for intubation without any significant effect on BPD or mortality [31]. Compared with NIPPV, it may lower the risk of BPD without any additional advantages. It has been relatively well-studied in post-extubation settings. A recent meta-analysis (11 trials, 1897 participants) suggested that NHFV, compared to NCPAP/NIPPV, may reduce the risk of extubation failure and is associated with lower BPD [31]. Further adequately powered studies exploring its efficacy and safety are needed before its routine clinical use.

Overall, the evidence supports the use of NIV as an effective initial and post-extubation neonatal ventilation strategy. The most crucial aspect of success is the timely and appropriate use of NIV. A written protocol for the initiation, titration, timely recognition of NIV failure, and weaning of NIV has been shown to be effective in reducing NIV failure. The success of NIV is influenced by various factors, including nursing care, GA, birth weight, severity of respiratory distress, surfactant use, antenatal steroid coverage, duration of invasive ventilation (in post-extubation scenarios), and nutritional status of the infant. There is little evidence of the best pressures to initiate support as well as criteria for upgrading or downgrading NIV. We have provided a table of the typical settings for various NIV supports in preterm infants (Table 2). Clinicians may use these settings as a practical tool but must individualize them according to infants’ GA and clinical status.

### Weaning of NIV

Weaning respiratory support is a combination of science and art [32]. A preterm infant should typically be weaned off from NIV when a set of predefined stability criteria are met. NIPPV is typically weaned off to NCPAP once the MAP is around 8 cm H_2_O, and the baby has good respiratory effort and muscle tone. In general, NCPAP weaning starts when the infant meets predefined criteria that include: the indication for starting respiratory support has resolved, absence of respiratory distress (respiratory rate < 60/min, no significant retractions), FiO_2_ < 0.25, no major apneas requiring intervention in the past 24 h, and the ability to tolerate brief periods of off-CPAP during routine care activities [32].

Several trials have compared various strategies and provided guidance on weaning strategies [32, 33]. Existing evidence suggests that interval weaning (periods off CPAP) compared to abrupt wean-off does not improve the chance of successful weaning, but may increase the NIV duration by 1.7 days (95% CI: 0.9 to 2.5) and hence should not be practiced [32]. Similarly, using HHHFNC for weaning from NCPAP does not improve weaning success but has the potential to increase the duration of NIV [33]. Therefore, the current evidence suggests that gradual weaning of NCPAP to a certain level (e.g. 5 cm H_2_O) and then abrupt weaning off to room air or low flow cannula (if the infant requires minimal O_2_ to maintain target saturations) is possibly the best strategy to wean preterm infants with RDS [33].

### Failure of NIV

**Fig. 1:**
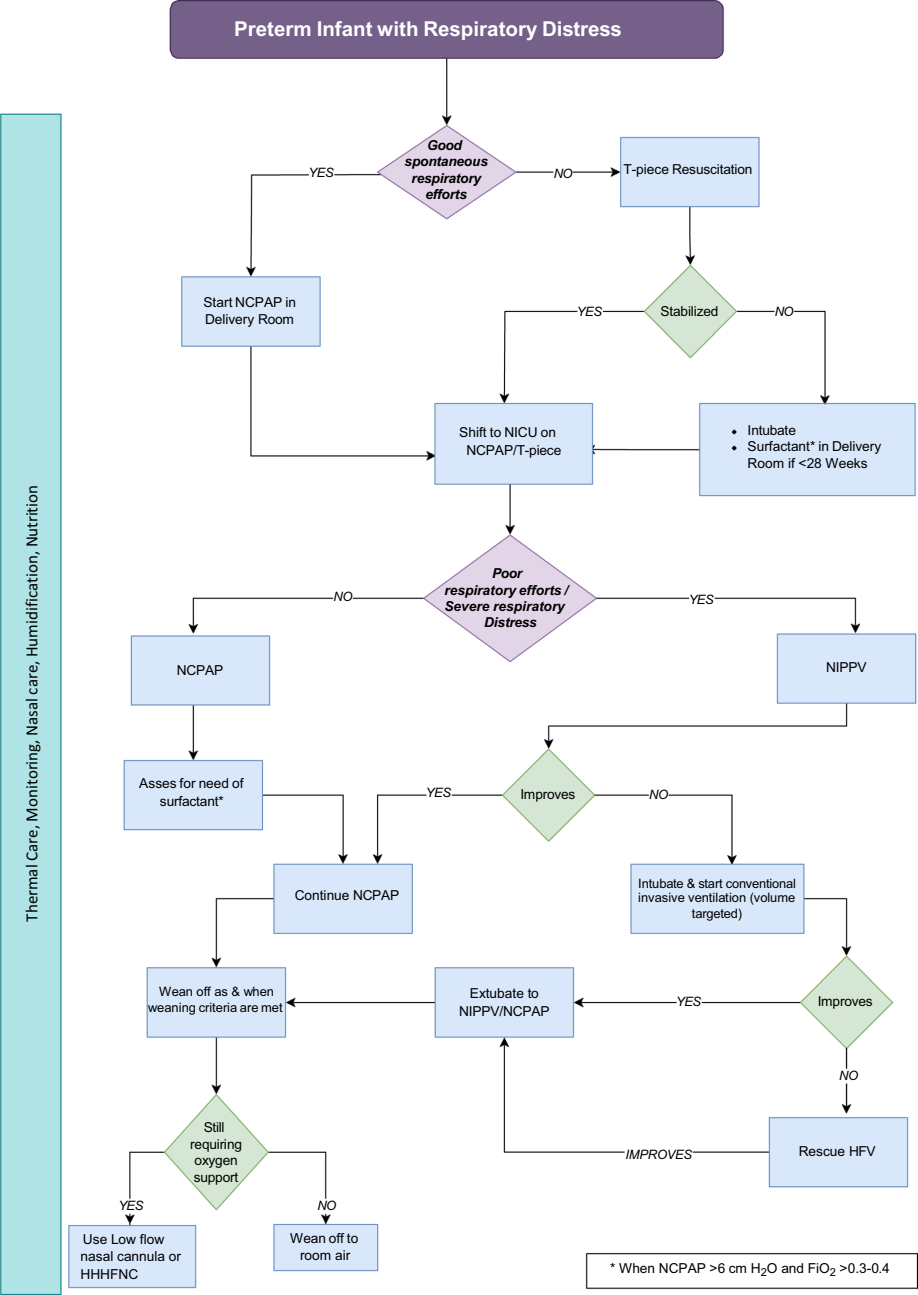
Suggested algorithm for an approach to an infant with respiratory distress

NCPAP or NIPPV failure is commonly defined as the occurrence of one or more of the following: frequent apnea (≥ 3 in 1 h in the previous 6 h), two or more episodes of severe apnea in 24 h requiring PPV, severe respiratory distress despite optimal NCPAP (Downes or Silverman Anderson Score > 6), acidosis (pH < 7.2), hypercarbia (pCO_2_ > 65 mmHg), and inability to maintain target saturation despite FiO_2_ of 0.60. The most common causes of NCPAP/NIPPV failure in developing countries such as India are delayed initiation of CPAP, lack of antenatal steroid use, delayed surfactant administration, nasal injury, lack of humidification, and lower CPAP pressures [34]. Good nursing care, appropriate choice of nasal interface, appropriate humidification, and timely initiation of NCPAP therapy can prevent many CPAP failures. If, despite correcting the above factors, infants still fail on NCPAP, a trial of NIPPV might be beneficial as it can help avert intubation. However, if the infant fails despite NIPPV or has severe acidosis, pulmonary hypertension, and/or hypoxia, timely intubation and IMV are the best options. We provided an algorithmic approach for choosing and titrating respiratory support (Fig. 1).

## Conclusions

In neonates with respiratory distress, NIV is the recommended first-line and post-extubation mode of support. In the DR, NCPAP or NIPPV using TPR is preferred. If surfactant is to be given, it is advisable to do so within the first 2 h of life in infants requiring NCPAP of 6–7 cm H_2_O and FiO_2_ > 0.3, using LISA or intubate, give surfactant, and extubate (InSurE). Infants should be transported to the NICU on positive pressure support by utilizing NCPAP or TPR. In the NICU, NIPPV is the preferred mode of NIV, especially in preterm neonates (if equipment and expertise are available), with NCPAP as the next best option. Nasal masks or SBP are the preferred nasal interfaces. HHHFNC is not recommended as a primary mode of NIV. Additional RCTs are needed to determine the optimal use of NHFV and NIV-NAVA modes of NIV. Box 2 provides the key recommendations for NIV strategies in neonates.

Box 1. Interventions to Prevent Nasal Injury in Infants Receiving Noninvasive Respiratory Support [14, 16, 17]

1. Use structured monitoring tools to periodically assess and document the nasal condition.
2. The nasal interface should be of appropriate size and should be used as per manufacturers’ recommendations.
3. Ensure adequate space between the nasal prong and nasal mucosa to prevent impingement and subsequent mucosal damage.
4. Nasal masks are preferred over short binasal prongs. If the unit is unable to implement universal and continuous use of nasal mask, alternation of short binasal prongs with nasal mask might be considered.
5. Use servo-controlled heated humidified gases in all infants receiving NCPAP/NIPPV.
6. Nasal barrier dressings (silicon/hydrocolloid) may be considered. These dressings should be cut in anatomical shape and should be regularly changed. They may impair visualization of early injury.
7. Encourage regular nasal massage with moisturizing oil.
8. Avoid excessive dryness or wetness around nostrils. If the nasal dressing is soiled or becomes moist, it should be immediately replaced. Saline drops may be instilled.
9. Regular audit of nasal injury.
10. Implement quality improvement approach to reduce nasal injury incidence.

Box 2. Key Messages

- In neonates with respiratory distress, noninvasive ventilation (NCPAP/NIPPV) should be used as a primary mode of ventilation.
- Nasal masks or short binasal prongs are the preferred nasal interfaces.
- If surfactant is to be given, it is advisable to do so within the first 2 h of life in infants requiring NCPAP of 6–7 cm H_2_O and FiO_2_ >0.3, using LISA or intubate, give surfactant and extubate (InSurE).
- Good nursing care and meticulous clinical examination are the keys to successful ventilation.

## Data Availability

The data used in this review are already in the public domain.
